# Work-related connectivity between Boston Logan international airport and urban communities with high social vulnerability during the COVID-19 pandemic

**DOI:** 10.1186/s40794-025-00249-0

**Published:** 2025-06-01

**Authors:** Daniel Begemann, Cristina Alonso, Samantha Bates, Edward T. Ryan, Allison T. Walker, Barry Keppard, Ann Marie Kissel, Flor Amaya, Sowmya R. Rao, Tyler S. Brown, Amir M. Mohareb, Julie H. Levison, Regina C. LaRocque

**Affiliations:** 1 Center of Complex Interventions, Cambridge, MA USA; 2 Division of Infectious Diseases, Massachusetts General Hospital, Boston, MA USA; 3 Department of Medicine, Harvard Medical School, Boston, MA USA; 4 Division of Global Migration Health, Centers for Disease Control and Prevention, Atlanta, GA USA; 5 Metropolitan Area Planning Council, Boston, MA USA; 6 City of Chelsea Public Health Division, Chelsea, MA USA; 7 Department of Global Health, Boston University School of Public Health, Boston, MA USA; 8 Section of Infectious Diseases, Boston Medical Center, Boston, MA USA; 9 Boston University Chobanian and Avedisian School of Medicine, Boston, MA USA; 10 Center for Global Health, Massachusetts General Hospital, Boston, MA USA; 11 Division of General Internal Medicine, Massachusetts General Hospital, Boston, MA USA; 12 Mongan Institute, Massachusetts General Hospital, Boston, MA USA

**Keywords:** Connectivity, Mobility, Social vulnerability, COVID-19

## Abstract

Airports may be high-risk sites for the spread of infectious diseases, including novel respiratory pathogens. While many studies have evaluated the higher burden of COVID-19 among essential workers, few studies have specifically analyzed the links between airport workers and surrounding communities during the COVID-19 pandemic. We used GPS-derived mobility data to estimate work- and travel-related visits from approximately 5000 Boston-area census block groups to Logan International Airport between January 2020 and August 2021. We stratified origin census block groups by their census tract-level Social Vulnerability Index and compared temporal trends and work-related airport visits across Social Vulnerability Index quartiles. Work-related visits to Boston Logan International Airport were more likely to originate from socially vulnerable communities (i.e., in the highest Social Vulnerability Index quartile), including cities that experienced disproportionately high rates of COVID-19 infection early during the COVID-19 pandemic (East Boston, Revere and Chelsea, Massachusetts). These differences persisted across the duration of the observation period. Our findings highlight higher social vulnerability and strong work-related mobility connections with an international travel hub as important overlapping risk factors in these urban communities. Protecting airport workers and their home communities from imported infectious diseases merits further attention as a public health priority.

**Supplementary Information:**

The online version contains supplementary material available at 10.1186/s40794-025-00249-0.

## Introduction

Airports are unique settings, in which travelers from diverse global sites interact closely with each other and with airport employees. Airline travel can facilitate the spread of respiratory infections among passengers and airline workers [1]. Higher volumes of airline passenger traffic were associated with higher rates of SARS-CoV-2 infection during the initial phases of the COVID-19 global pandemic [2]. Moreover, U.S. counties containing international airports had a higher incidence rate of infection in initial months of the COVID-19 pandemic [3]. Airports are linked to local communities through a variety of ways, including travelers, employees, vendors, and contractors.

In the first year of the COVID-19 pandemic, a disproportionate burden of infection and mortality was evident in socially vulnerable communities, including populations with housing instability, fewer economic opportunities, and poor access to healthcare resources [4]. COVID-19 burden in these communities may be attributable to several social determinants of health, including a preponderance of so-called “essential workers,” many of whom incurred occupational exposures to SARS-CoV-2 without adequate protections [5]. For example, public transit workers in California experienced COVID-19 incidence and mortality rates that were 1.5 times as high as in other industries [6, 7]. In Massachusetts, communities with a high proportion of essential workers and ethnic minorities had between 15 and 30% increased COVID-19 incidence rates in 2020 [4]. 

Mapping work-related mobility between airports and their surrounding communities can help elucidate linkages to socially vulnerable communities who may need additional support before, during, or after infectious diseases outbreaks– particularly for infections that may spread by international travel [3, 8]. However, there is limited information about the communities where airport workers live and social determinants of health in these communities. Our objective was to estimate work-related trips between Boston-area communities with high social vulnerability and Boston Logan International Airport, an international transportation hub and New England’s largest airport, during the initial phase of the COVID-19 pandemic.

## Methods

### Study design

We conducted an ecological study to estimate the distribution of communities with airport workers in the Greater Boston area during the first eighteen months of the COVID-19 pandemic. We mapped estimates of the number of airport workers in each census block group across the Greater Boston Area and compared them with the Centers for Disease Control and Prevention (CDC) Social Vulnerability Index, a composite marker of socioeconomic status, household composition, minority status, and vehicle access [9]. Past studies have shown the close association between Social Vulnerability Index and COVID-19 burden during this period of the pandemic [10]. 

### Mobility data

We used aggregated, anonymized GPS-derived location data from mobile phone applications collected in Massachusetts from January 2020– August 2021, obtained from SafeGraph (https://www.safegraph.com/). The dataset includes information about travel to and from census block groups and points of interest, such as airports, hotels, and restaurants. The dataset includes “dwell time buckets,” indicating the amount of time spent at each point of interest and counts of visitors between specific census block groups and specific points of interest. Prior research has validated the accuracy of SafeGraph data by comparing it with Google mobility data in the US and observing matching trends [11–13]. 

### Social vulnerability index data

The CDC’s Agency for Toxic Substances and Disease Registry (ATSDR) assigns a Social Vulnerability Index score to each census tract, ranging from 0 (lowest vulnerability) to 1 (highest vulnerability) [9]. Each census block group in our SafeGraph dataset belongs to a census tract, so we assigned census tract-based Social Vulnerability Index scores to all census block groups within its boundaries. We grouped Social Vulnerability Index scores into one of three categories for each census block group: Social Vulnerability Index 0.00–0.50, Social Vulnerability Index 0.50–0.75, and Social Vulnerability Index 0.75–1.00.

### Analysis of “visits for work” and “visits for travel” to Boston Logan international airport

According to the International Air Transport Association Global Passenger Survey, most travelers spend three or less hours at the airport [14]. Based on this, we defined any visit to one of the 134 points of interest located in census block groups within or contiguous to Logan Airport that lasted for four or more hours as a “visit for work” and any visit that lasted less than four hours as a “visit for travel.” We normalized the raw counts to points of interest at Logan International Airport based on the population of each evaluated census block group to avoid over-weighting travel frequency between Logan International Airport and census block groups with large populations (Supplemental Methods). We used the normalized count data to calculate the number of “visits for work” and “visits for travel” to Logan International Airport for each census block group. We used a one-sided t-test to compare the number of airport visits from communities across different Social Vulnerability Index quartiles.

### Ethics approval

we used commercially and publicly available data without any individual identifiers, and we obtained ethics approval for this study from the Mass General Brigham institutional review board, which deemed the project to be non-human subjects research.

### Patient and public involvement

No patients were involved in this study. Community involvement in this study occurred in the design, implementation, interpretation, and dissemination process through community volunteers for the Center for Complex Interventions, the Metropolitan Area Planning Council of Boston, and local boards of public health.

## Results

After normalization, we identified 783,006 visits to a point of interest in or immediately contiguous to Boston Logan International Airport during the study period: 61,074 (8%) were visits for work and 721,932 (92%) were visits for travel. Visits for work remained relatively consistent during the study period, whereas visits for travel fluctuated, with a nadir observed in April 2020 when numerous international travel restrictions were enacted (Supplemental Figure S1).

Of the 61,074 visits for work during the study period, 22,110 (36%) originated from census block groups with Social Vulnerability Indices between 0.75 and 1.00; 14,031 (23%) originated from census block groups with Social Vulnerability Indices between 0.50 and 0.75; 12,756 (21%) originated from census block groups with Social Vulnerability Indices between 0.25 and 0.50; and 12,175 (20%) originated from census block groups with Social Vulnerability Indices between 0 and 0.25. Significantly more visits for work arose from census block groups in each of the top two Social Vulnerability Index quartiles than from census block groups in the lowest two Social Vulnerability Index quartiles (Figure). This difference persisted across the entire duration of the dataset (Supplemental Figure S2.)

**Fig. 1:**
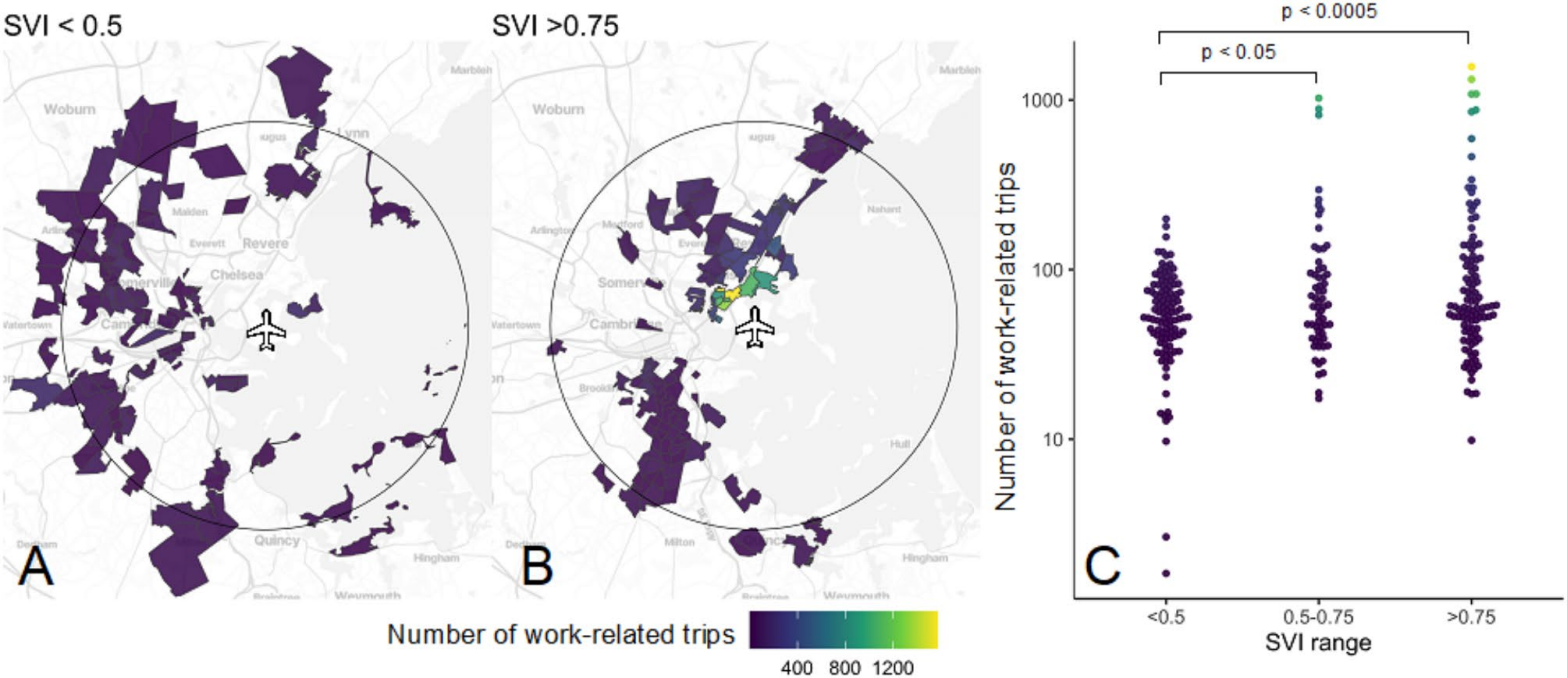
Estimated number of work-related trips to Boston Logan Airport by origin census tract Social Vulnerability Index. (**A**) Total work-related trips (January 2020 - August 2021) originating from census tracts in the lowest Social Vulnerability Index quartile (least vulnerable). (**B**) Total work-related trips originating from census tracts in the highest Social Vulnerability Index quartile (most vulnerable). Circle shows a 7.5 mile radius around Boston Logan Airport. (**C**) Comparison of census tract-level total work-related trips (log-transformed y-axis) by Social Vulnerability Index quartile. Each point represents a unique census tract. P-values comparing trip totals by Social Vulnerability Index quartile were calculated using a one-sided t-test. Census tracts in (**A**) and (**B**), and points in (**C**) are colored according to the total number of work-related trips

We mapped visits for work from communities with the highest quartile of Social Vulnerability Index score (> 0.75) and those with the two lowest quartiles of Social Vulnerability Index scores (< 0.5). We include a circle marking a 7.5-mile radius around Boston Logan International Airport, which corresponds to the approximate extent of the Boston metro area public transportation system [15], excluding long distance commuter train lines (Fig. 1). Of the 22,110 visits for work from high- Social Vulnerability Index locations (Social Vulnerability Index > 0.75) during the study period, 15,477 (70%) originated in census block groups within this radius; census block groups with the highest total number of work-related trips were located in East Boston, Revere and Chelsea, Massachusetts.

## Discussion

International travel plays an important role in the global spread and amplification of infectious diseases, including respiratory pathogens [1]. Essential workers experienced high rates of infection during the early phase of the COVID-19 pandemic, as did individuals residing in communities with high social vulnerability [4, 5]. Our analysis identifies strong work-related mobility links between an international travel hub in Boston, Massachusetts, and adjacent communities with high social vulnerability indices during the COVID-19 pandemic. These gateway communities, including Chelsea, Revere and East Boston, are home to many immigrants and low-wage workers, many of whom live in multigenerational housing and who experience higher rates of chronic diseases and food insecurity [16]. These communities experienced disproportionate impacts during COVID-19, compared to others in the Boston area, particularly in the early period of the pandemic [17, 18]. 

Our study is unique in applying observed mobility data to identify an empiric link between an airport and neighboring communities– a link which may have facilitated amplification and spread of the SARS CoV-2 respiratory virus. The persistence of work-related airport visits during travel restrictions, and the corresponding sharp decline in travel-related visits, supports our assertion that GPS-derived mobility data can differentiate work- versus travel-related airport trips. Nevertheless, our study design has limitations. We applied a data-informed but still heuristic definition to separate visits for travel and visits for work, and we may have misclassified trips. While this descriptive study generates an important hypothesis that airport workers may facilitate COVID-19 transmission between transportation centers and socially vulnerable communities, this association is still subject to possible confounding that our study is not well-positioned to examine. The ecologic trends we observed may not be generalizable to other airports or transit hubs, and analyses of Social Vulnerability Index mobility trends at other major transit sites are warranted.

This is the first study to our knowledge that illustrates the scope of mobility connections between socially vulnerable communities and an international travel hub. Protecting airport workers and consequently their home communities from imported infectious diseases merits further attention as a public health priority; such interventions could include airport ventilation upgrades, low-barrier infectious disease testing programs, occupational vaccination, and paid sick leave.

## Electronic supplementary material

Below is the link to the electronic supplementary material.

Supplementary Material 1

## Data Availability

All data used in this study are commercially and publicly available.

## References

[1] Mangili A, Gendreau MA. Transmission of infectious diseases during commercial air travel. Lancet. 2005;365(9463):989–96. 10.1016/S0140-6736(05)71089-8. 15767002 10.1016/S0140-6736(05)71089-8 PMC7134995

[2] Oztig LI, Askin OE. Human mobility and coronavirus disease 2019 (COVID-19): a negative binomial regression analysis. Public Health. 2020;185:364–7. 10.1016/j.puhe.2020.07.002. 32739776 10.1016/j.puhe.2020.07.002 PMC7351378

[3] Chokshi A, DallaPiazza M, Zhang WW, Sifri Z. Proximity to international airports and early transmission of COVID-19 in the united States-An epidemiological assessment of the geographic distribution of 490,000 cases. Travel Med Infect Dis. 2021;40:102004. 10.1016/j.tmaid.2021.102004. 33640475 10.1016/j.tmaid.2021.102004 PMC7906855

[4] Tieskens KF, Patil P, Levy JI, et al. Time-varying associations between COVID-19 case incidence and community-level sociodemographic, occupational, environmental, and mobility risk factors in Massachusetts. BMC Infect Dis. 2021;21:686. 10.1186/s12879-021-06389-w. 34271870 10.1186/s12879-021-06389-w PMC8283097

[5] Haley BM, Patil P, Levy JI, et al. Evaluating COVID-19 risk to essential workers by occupational group: a case study in Massachusetts. J Community Health. 2024;49:91–9. 10.1007/s10900-023-01249-x. 37507525 10.1007/s10900-023-01249-x PMC10823035

[6] Chen YH, Riley AR, Duchowny KA, et al. COVID-19 mortality and excess mortality among working-age residents in California, USA, by occupational sector: a longitudinal cohort analysis of mortality surveillance data. Lancet Public Health. 2022;7(9):e744–53. 10.1016/S2468-2667(22)00191-8. 36057273 10.1016/S2468-2667(22)00191-8 PMC9433054

[7] Heinzerling A, Vergara XP, Gebreegziabher E, et al. COVID-19 outbreaks and mortality among public transportation workers — California, January 2020–May 2022. MMWR Morb Mortal Wkly Rep. 2022;71:1052–6. 10.15585/mmwr.mm7133a4. 35980867 10.15585/mmwr.mm7133a4 PMC9400527

[8] Huber C, Watts A, Grills A, et al. Modelling airport catchment areas to anticipate the spread of infectious diseases across land and air travel. Spat Spatiotemporal Epidemiol. 2021;36:100380. 10.1016/j.sste.2020.100380. 33509428 10.1016/j.sste.2020.100380 PMC10413988

[9] Centers for Disease Control and Prevention/ Agency for Toxic Substances and Disease Registry. / Geospatial Research, Analysis, and Services Program. CDC/ATSDR Social Vulnerability Index 2020 Database Massachusetts. https://www.atsdr.cdc.gov/placeandhealth/svi/data_documentation_download.html. Accessed on January 10, 2025.

[10] Khazanchi R, Beiter ER, Gondi S, Beckman AL, Bilinski A, Ganguli I. County-Level association of social vulnerability with COVID-19 cases and deaths in the USA. J Gen Intern Med. 2020;35(9):2784–7. 10.1007/s11606-020-05882-3. 32578018 10.1007/s11606-020-05882-3 PMC7311112

[11] Chang S, Pierson E, Koh PW, et al. Mobility network models of COVID-19 explain inequities and inform reopening. Nature. 2021;589(7840):82–7. 10.1038/s41586-020-2923-3. 33171481 10.1038/s41586-020-2923-3

[12] Jay J, Bor J, Nsoesie EO, et al. Neighbourhood income and physical distancing during the COVID-19 pandemic in the united States. Nat Hum Behav. 2020;4(12):1294–302. 10.1038/s41562-020-00998-2. 33144713 10.1038/s41562-020-00998-2 PMC8107986

[13] Klise K, Beyeler W, Finley P, Makvandi M. Analysis of mobility data to build contact networks for COVID-19. PLoS ONE. 2021;16(4):e0249726. 10.1371/journal.pone.0249726. 33857208 10.1371/journal.pone.0249726 PMC8049304

[14] International Air Transport Association. Annual Review 2021 77th Annual General Meeting, Boston USA. October 2021. https://www.iata.org/contentassets/c81222d96c9a4e0bb4ff6ced0126f0bb/iata-annual-review-2021.pdf. Accessed January 13, 2025.

[15] Massachusetts Bay Transportation Authority. Boston area public transit map. Available from: https://cdn.mbta.com/sites/default/files/2024-12/2024-12-15-system-map_0.pdf. Accessed 14 January 2025.

[16] Alonso C, Keppard B, Bates S, Cortez D, Amaya F, Dinakar K. The Chelsea project: turning research and wastewater surveillance on COVID-19 into health equity action, Massachusetts, 2020–2021. Am J Public Health. 2023;113(6):627–30. 10.2105/AJPH.2023.307253. 37023385 10.2105/AJPH.2023.307253 PMC10186821

[17] Naranbhai V, Chang CC, Beltran WFG, et al. High Seroprevalence of anti-SARS-CoV-2 antibodies in Chelsea, Massachusetts. J Infect Dis. 2020;222(12):1955–9. 10.1093/infdis/jiaa579. 32906151 10.1093/infdis/jiaa579 PMC7499676

[18] Brown T, de Salazar Munoz PM, Bhatia A, et al. Geographically skewed recruitment and COVID-19 Seroprevalence estimates: a cross-sectional serosurveillance study and mathematical modelling analysis. BMJ Open. 2023;13(3):e061840. 10.1136/bmjopen-2022-061840. 36882240 10.1136/bmjopen-2022-061840 PMC10008195

