# Supplementary material for: Work-related connectivity between Boston Logan international airport and urban communities with high social vulnerability during the COVID-19 pandemic
Source: Trop Dis Travel Med Vaccines. 2025 Jun 1;11:15. doi: 10.1186/s40794-025-00249-0 (PMC12126863; doi:10.1186/s40794-025-00249-0)
Supplement: Supplementary file 1 — Supplementary Material 1 [file 40794_2025_249_MOESM1_ESM.docx]

**Supplement to:**

Worked-related connectivity between Boston Logan International Airport and communities with high social vulnerability during the COVID-19 epidemic


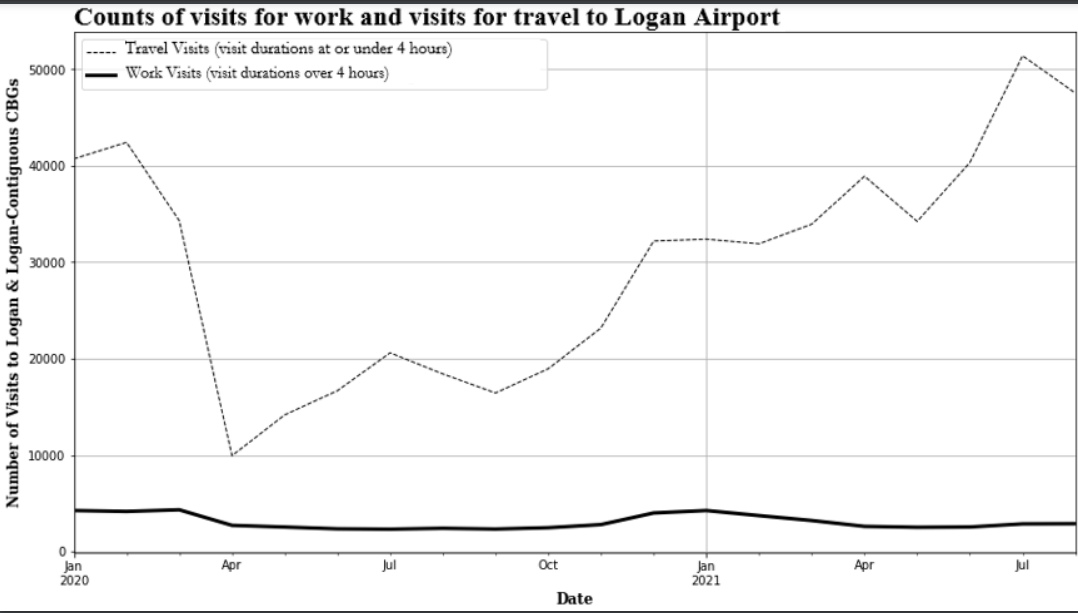


**Supplemental Figure 1:** Visits for work and visits for travel to Boston Logan International Airport, January 2020 – August 2021


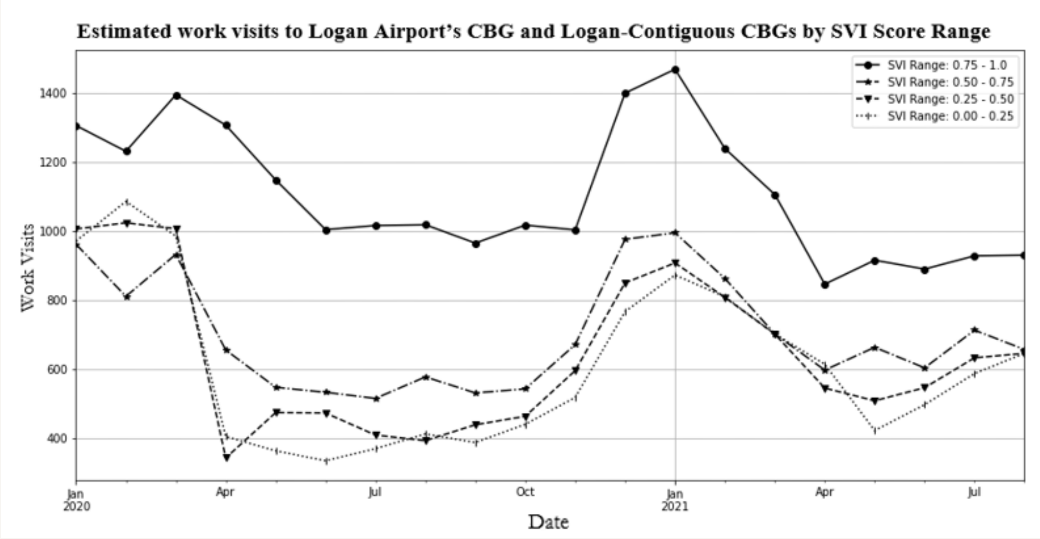


**Supplemental Figure 2:** Visits for work to Boston Logan International Airport by SVI score range, January 202 – August 2021
